# Genomic characteristics and drug resistance phenotype of *Levyella massiliensis* isolated from blood

**DOI:** 10.1186/s12866-026-04967-z

**Published:** 2026-03-24

**Authors:** Tao Gu, Ying Liu, Jian Zhou, Baojian Liu, Xiaoyu Wei, Yong Hu, Yu Wang, Shijun Li

**Affiliations:** 1 Key Laboratory of Microbio and Infectious Disease Prevention and Control in Guizhou Province, Guizhou Center for Disease Control and Prevention, China, Guiyang, Guizhou 550004 China; 2 Key Laboratory of Environmental Pollution Monitoring and Disease Control, Ministry of Education, School of Public Health, Guizhou Medical University, Guiyang, Guizhou 561113 China; 3 The First People’s Hospital of Guiyang, Guiyang, Guizhou 550002 China

**Keywords:** *Levyella massiliensis*, Bacteremia, Complete genome sequence, Multidrug resistance, Comparative genomics

## Abstract

This study reports the first isolation of a strain of *Levyella massiliensis* (strain HGL1) from human blood samples in China. Currently, the genomic characteristics, antibiotic resistance, and pathogenic potential of this species have not been fully studied. To systematically decipher the phylogenetic position, genomic composition, and potential pathogenicity-related factors of strain HGL1, this study employed a dual-platform sequencing strategy (PacBio Sequel II and Illumina NovaSeq PE150) for whole-genome sequencing of strain HGL1 and assessed its antibiotic susceptibility through antimicrobial susceptibility testing. Phylogenetic analysis revealed that strain HGL1 and *L. massiliensis* D22-N-7-79 reside within the same evolutionary clade, with an Average Nucleotide Identity (ANI) of 97.88% and a digital DNA-DNA hybridization (dDDH) value of 80%, supporting the classification of strain HGL1 as a member of *L. massiliensis*. Genomic analysis indicated that the HGL1 genome is 1,682,293 bp in size with a GC content of 48.85%, harboring a total of 1,613 predicted coding genes, 56 potential virulence genes, and 4 antibiotic resistance genes. Antimicrobial susceptibility testing results showed that this bacterium is susceptible to *tigecycline*, *amoxicillin/clavulanic* acid, and some *β-lactam antibiotics*; however, it exhibits resistance to macrolides (e.g., *erythromycin*, *clarithromycin*, *azithromycin*), *trimethoprim*/*sulfamethoxazole*, third-generation cephalosporins (*ceftriaxone*, *cefotaxime*), quinolones (*ciprofloxacin*, *levofloxacin*), and *gentamicin*. These findings demonstrate that *L. massiliensis* HGL1 is a bacterium possessing multidrug resistance and potential human pathogenicity. In-depth analysis of its genomic characteristics and resistance phenotype provides a scientific basis for understanding the biology and combating infections caused by this rare pathogen, underscoring the necessity for enhanced surveillance and research on this bacterium in future clinical practice.

## Introduction

Anaerobic infections are a common type of severe clinical infection that can progress to bacteremia and are often associated with polymicrobial infections. In previous clinical practice, *Bacteroides fragilis* has been regarded as the primary pathogen in anaerobic infections [1], however, with the widespread application of MALDI-TOF MS technology in clinical microbiology laboratories [2, 3], an increasing number of atypical anaerobic microorganisms have been identified as infectious pathogens. These pathogens are associated with various clinical infection types such as respiratory tract infections and bloodstream infections [4, 5].

*L. massiliensis* is a relatively rare Gram-positive anaerobic bacterium, first described by La Scola et al. in 2010, with its type strain CSUR P128^T^ isolated from a human blood sample [6]. To date, reports concerning this bacterium remain limited. Apart from the type strain, its detection has primarily relied on culture-independent metagenomic sequencing techniques. For instance, one study detected this bacterium via metagenomic sequencing in scrotal fluid from a patient following inguinal hernia surgery [7]; another metagenomic sequencing study on body fluids from leukemia patients also identified L. massiliensis, suggesting a potential symbiotic relationship with the gut microbiota in acute leukemia patients [8]. Prior to this study, no complete genome sequence of *L. massiliensis* was available in public databases, which has hindered comprehensive genomic characterization. Systematic studies on the genomic features, pathogenic potential, and detailed antimicrobial resistance profile of this bacterium are still lacking. Furthermore, the lack of reliable reference spectra for this bacterium in the clinically widely used MALDI-TOF mass spectrometry also highlights the limitations of conventional diagnostic techniques in identifying *L. massiliensis*.

In China, this study successfully isolated and cultured a strain of *L. massiliensis* from a blood sample of a male patient for the first time. Given the current lack of comprehensive genomic and functional analyses for *L. massiliensis*, we employed a dual-platform sequencing strategy for the first time to determine the first complete genome of *L. massiliensis*, with the aim of elucidating its genomic structure and functional potential through multi-database annotation, and to describe the morphological and phylogenetic relationships of this bacterium. This study aims to characterize *L. massiliensis*, a rare anaerobic bacterium, and to provide foundational data that may inform understanding of infections associated with this organism.

## Method

### Isolation, cultivation, and DNA extraction of strain HGL1

The HGL1 strain was isolated from a blood sample of a hospitalized patient in a hospital in Guizhou Province, China. The blood samples were inoculated onto blood agar plates and cultured under aerobic, anaerobic, and microaerophilic conditions. After 3-day incubation at 37 °C with 5% CO₂ under anaerobic conditions, pinpoint colonies were observed and designated as HGL1. The strain was preserved in 20% glycerol stock vials and stored long-term at -80 °C. Genomic DNA was extracted using bacterial DNA extraction kits from Hangzhou Baiyi Technology Co. Ltd (Magnetic Bacteria Genomic DNA Kit (RBJ1015)). The DNA was eluted with 60 µl elution buffer and stored at -80 °C for further use.

### 16 S rRNA gene sequencing and identification

For rapid preliminary identification of the isolate, the 16 S rRNA gene was amplified and sequenced via the Sanger method. Subsequently, upon completion of whole-genome sequencing, the full-length 16 S rRNA gene sequence was extracted from the assembled genome for definitive phylogenetic analysis and species confirmation. Bacterial 16 S rRNA gene was amplified using universal primers 27 F (5′-AGAGTTTGATCCTGGCTCAG-3′) and 1492R (5′-GGTTACCTTGTTACGACTT-3′) with genomic DNA as template. The 50-µL PCR system comprised: 25 µL Taq Premix (TaKaRa), 2 µL each primer (27 F/1492R), 2 µL DNA template, and 19 µL nuclease-free water. Thermal cycling protocol: Initial denaturation at 94 °C (5 min); 35 cycles of denaturation (94 °C, 15 s), annealing (50 °C, 30 s), extension (72 °C, 90 s); final extension (72 °C, 5 min). Amplified products were verified by 1% agarose gel electrophoresis and sent for bidirectional sequencing at Tsingke Biotechnology Co., Ltd (Chongqing). The obtained sequences were aligned using the BLASTn algorithm against the NCBI rRNA/ITS databases(16 S ribosomal RNA sequences (Bacteria and Archaea)) for preliminary taxonomic identification.

### Whole genome sequencing and assembly

To combine the high accuracy of second-generation sequencing (Illumina) with the long-read advantage of third-generation sequencing (PacBio) [9, 10], this study employed a hybrid sequencing assembly strategy to assemble the sequencing data of strain HGL1 [11, 12]. The purity and integrity of the extracted DNA were assessed by agarose gel electrophoresis, and quantification was performed using Qubit. Illumina library preparation and quality control: DNA samples passing electrophoresis were randomly sheared into fragments of approximately 350 bp using a Covaris ultrasonicator. The sheared DNA fragments were then processed using the NEBNext^®^ Ultra™ DNA Library Prep Kit for Illumina (NEB, USA) to complete library preparation through steps including end repair, A-tailing, adapter ligation, purification, and PCR amplification. After library construction, preliminary quantification was performed using Qubit 2.0, and the library was diluted to 2 ng/µL. The insert size was then checked using an Agilent 2100 Bioanalyzer. Once the insert size met expectations, the effective library concentration was accurately quantified via qPCR to ensure library quality. PacBio library preparation: The SMRTbell™ Template Prep Kit 2.0 was used to construct SMRTbell libraries. Qualified DNA was sheared to the desired size using Covaris g-TUBEs, followed by DNA damage repair and end repair. Hairpin adapters were then ligated to both ends of the DNA fragments using DNA ligase. The fragments were purified using AMPure PB beads. Size selection was performed using the Blue Pippin system, and the SMRTbell library was concentrated using AMPure PB beads. Subsequent steps included additional DNA damage repair and another round of purification with AMPure PB beads. The final library was quantified using Qubit, and the insert size distribution was analyzed on an Agilent 2100 Bioanalyzer. Upon passing quality control, the libraries were sequenced on the Illumina NovaSeq (PE150) and PacBio Sequel II/Sequel IIe platforms according to their effective concentrations and the desired data yield. Based on the obtained sequencing data, the reads were assembled using Canu software (version 2.0; https://github.com/marbl/canu/*)* to generate a preliminary assembly reflecting the basic genomic features of the sample. This assembly was then polished in three rounds using Racon (version 1.4.13) with the PacBio reads, followed by three additional rounds of polishing using Pilon (version 1.22) with the Illumina reads, yielding the final assembly.

### Similarity of whole genomes

This study evaluated whole-genome similarity using Average Nucleotide Identity (ANI, http://www.anicalculator/ezbiocloud.net*)* and in silico DNA-DNA hybridization (DDH). ANI serves as a key indicator of genetic distance, with 95% generally adopted as the species delineation threshold [13]; whereas digital DDH values were determined via the GGDC 3.0 online intergenomic distance calculator (http://ggdc.dsmz.de/ggdc.php*)* [14], applying 70% as the species demarcation benchmark. The 16 S rRNA gene sequence of strain HGL obtained through whole-genome sequencing was subjected to BLAST analysis against the NCBI database to identify homologous sequences from closely related type strains. Phylogenetic tree construction was performed using the Neighbor-Joining method in MEGA 12.0 software [15].

### Gene prediction, annotation and comparison analysis

Protein-coding genes were predicted in the newly sequenced genome using GeneMarkS (Version 4.17) (http://topaz.gatech.edu/GeneMark/*)* [16]. tRNA prediction was performed by tRNAscan-SE (v1.3.1) with default parameters for bacterial genomes. rRNA genes were predicted with rRNAmmer (Version 1.2). For predicting sRNAs, the first step is to perform an alignment and annotation using the Rfam database, followed by determining the final sRNAs using the cmsearch program (version 1.1rc4) with default parameters [17, 18]. Prophages in the sample genome were predicted using phiSpy (Version 2.3). Based on the annotation results, a genome circle diagram including features such as GC content, gene density, and non-coding RNA distribution was drawn using Circos [19].

Protein sequences encoded by the genome were analyzed using SignalP 4.1 for signal peptide prediction and TMHMM 2.0c for transmembrane domain identification, with integrated assessment of secretory protein potential. Using the Diamond alignment algorithm (e-value cutoff ≤ 1 × 10 − 5), predicted protein sequences were systematically compared against authoritative databases including NR, GO, KEGG, COG, TCDB, CAZY, PHI, Pfam and Swiss-Prot for functional annotation. For each protein sequence, functional annotations were filtered based on highest alignment scores (default parameters: sequence identity ≥ 40%, coverage ≥ 40%). Protein-coding sequences from HGL1 strain were comparatively analyzed with both the Virulence Factors Database (VFDB) and the Comprehensive Antibiotic Resistance Database (CARD). Integration of gene sequences with corresponding virulence factors and antibiotic resistance gene functions yielded systematic annotations of virulence and resistance genes.

### Phenotypic analysis of antibiotic resistance

Experiments were conducted in accordance with the latest guidelines from the Clinical and Laboratory Standards Institute (CLSI 2024, 34th edition) [20]. Quality control strains included *Bacteroides fragilis* ATCC 25,285 for anaerobic conditions and *Clostridioides difficile* ATCC 700,057 for fastidious organism requirements. E-test strips (bioMérieux) were employed for antibiotic susceptibility testing with predefined concentration gradients. The E-test method combines the technical principles of both agar diffusion and broth dilution methods. It generates continuous and stable antibiotic concentration gradients on Brucella blood agar plates. After 48 h of anaerobic incubation, the elliptical inhibition zones around the strips were measured. The MIC value was determined at the intersection point between the inhibition zone edge and the strip’s concentration scale.

## Result

### Basic characteristics of strain HGL1

**Fig. 1:**
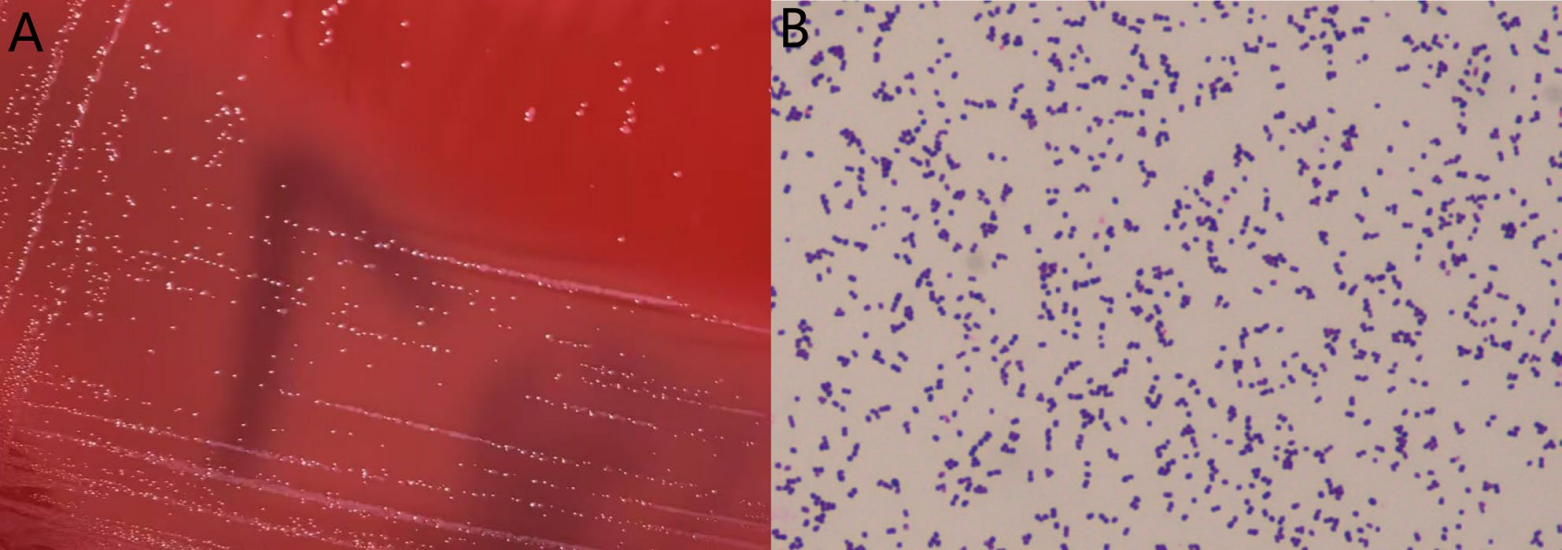
**A** Colony morphology after growing on the blood AGAR plate for 72 h. **B** Observation under a Gram staining microscope

The isolated and purified strain HGL1 was inoculated onto blood agar plates and cultured at 37℃ under anaerobic conditions (80% N₂, 10% H₂, 10% CO₂) for 72 h. Cultivation results showed small colonies on blood agar with diameters of 0.5–1 mm, low convex, circular, entire margins, smooth and moist surfaces, and grayish-white coloration (Fig. 1A). Gram staining revealed Gram-positive coccus with regular morphology and no spore formation (Fig. 1B).

### Identification of strain HGL1

**Fig. 2:**
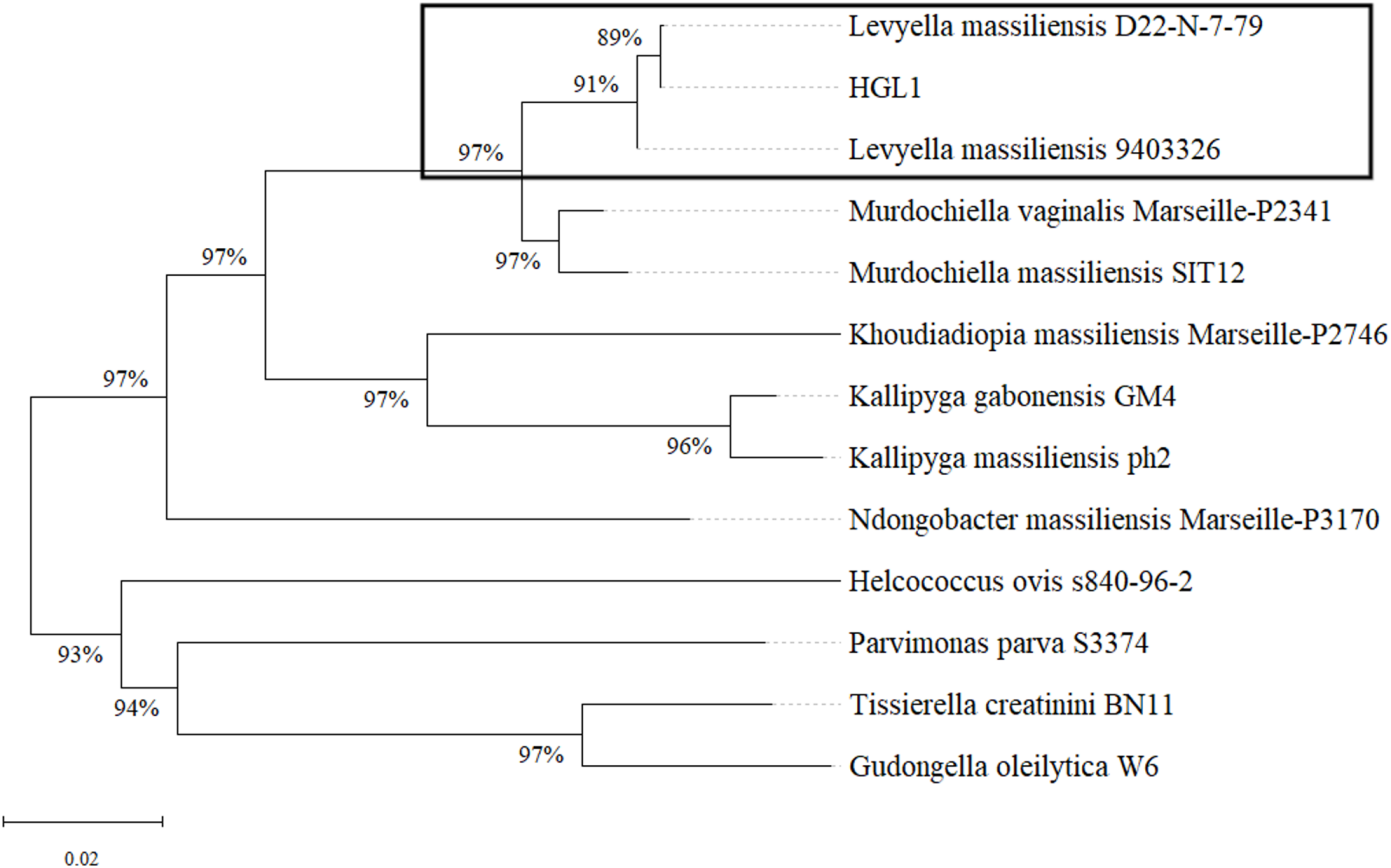
The phylogenetic tree (Neighbour-joining) of the 16 S rRNA gene sequence of strain HGL1

The 16 S rRNA gene sequence of strain HGL1 was obtained through whole genome sequencing, resulting in a sequence of 1,370 bp in length. This sequence has been submitted to the GeneBank database. NCBI blastn analysis revealed that strain HGL1 showed 99.93% and 99.71% sequence similarity to *L. massiliensis* strains D22-N-7-79 (PQ222901.1) and 9,403,326 (NR_133039.1), respectively. The 16 S rRNA gene sequences of closely related strains (showing high similarity in BLAST analysis) were retrieved, and a multiple sequence alignment was generated using the ClustalW algorithm implemented in MEGA12 software. Then, a phylogenetic tree was then constructed using the Neighbor-Joining (NJ) method, as shown in Fig. 2. Strain HGL1 was preliminarily identified as *L. massiliensis* based on > 98.7% 16 S rRNA similarity (species delineation threshold) and its clustering with type strains in a highly supported clade (Bootstrap > 70%). To further confirm the classification status, the *L. massiliensis* strain 9,403,326 (Although strain D22-N-7-79 showed the highest 16 S rRNA similarity, its complete genome is not available in public databases. Therefore, we selected *L. massiliensis* strain 9403326 as the reference, as it possesses a complete genome assembly and represents a phylogenetically close relative) that has the closest phylogenetic relationship was selected as the reference strain, and the average nucleotide identity (ANI) and digital DNA-DNA hybridization (dDDH) analyses were conducted on the entire genome. Comparative genomic analysis demonstrated 97.88% average nucleotide identity (ANI) between strain HGL1 and *L. massiliensis* strain 9,403,326; Digital DNA-DNA hybridization (dDDH) values were estimated using the Genome-to-Genome Distance Calculator (GGDC) 3.0, which employs three distinct comparison models: Formula 1 (based on sequence identity only), Formula 2 (considering identity and high-scoring segment pair length), and Formula 3 (a more complex model also incorporating G + C content). The resulting dDDH values were 73.4%, 80%, and 77.2% for Formulas 1, 2, and 3, respectively. The associated confidence probabilities that the true DDH value is ≥ 70% (the species threshold) were 81.68%, 90.58%, and 92.29%, respectively. These molecular characteristics support the identification of strain HGL1 as a member of the *L. massiliensis* species.

### Genomic characteristics

**Fig. 3:**
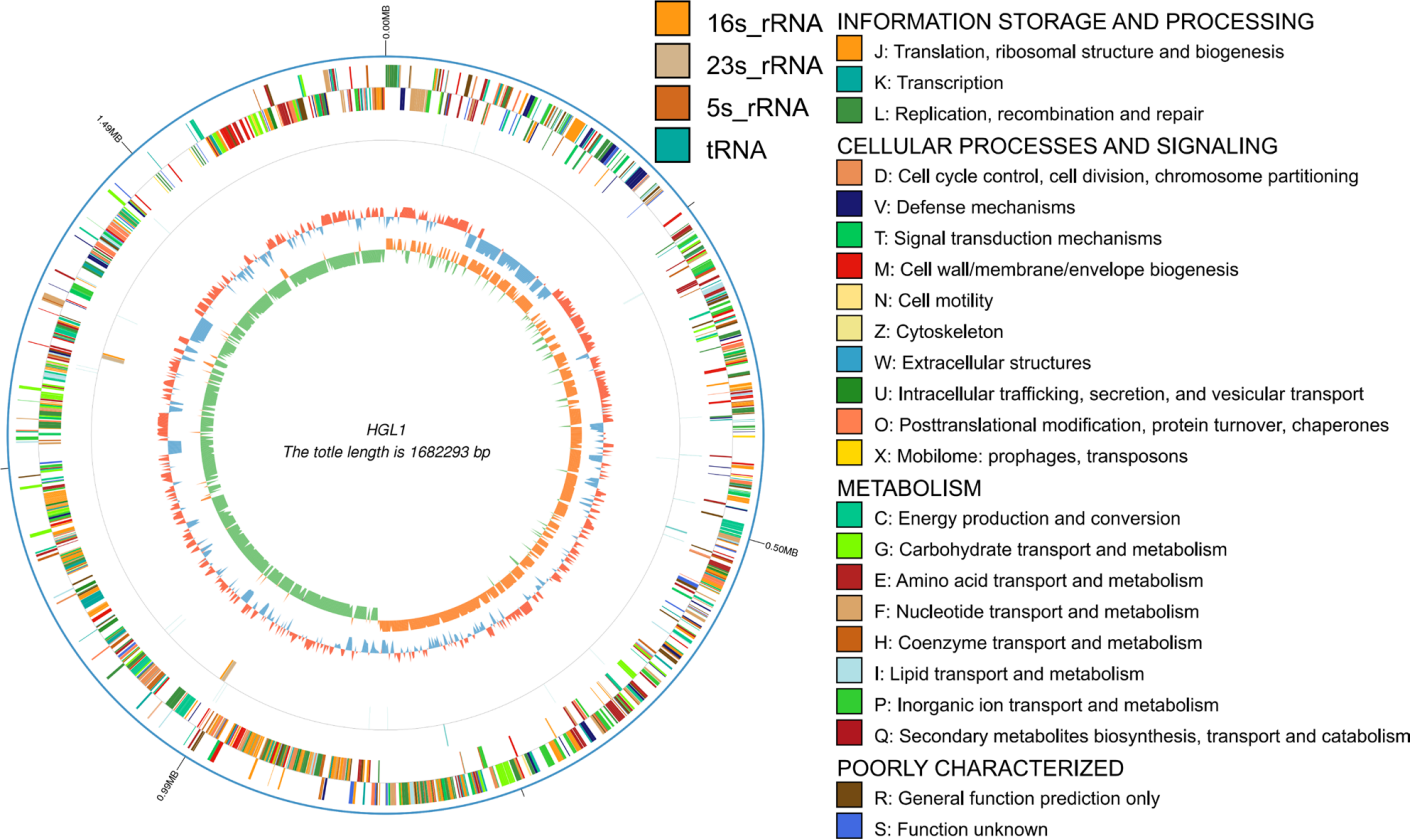
Genome-wide map of strain HGL1

After the genome sequencing data were assembled, the complete genome sequence of the HGL1 strain was obtained, which was 1,682,293 bp long, with a GC content of 48.85%. Genome annotation identified a total of 1,613 coding genes, with an average gene length of 928 bp. The coding region covered 89.97% of the entire genome. In the non-coding RNA analysis, a total of 47 tRNA genes and 6 rRNA genes were identified. Among these, the 6 rRNA genes comprise two 5 S rRNA, two 16 S rRNA, and two 23 S rRNA genes. Prophages are nucleic acid fragments of temperate phages integrated into host genomes, capable of replicating synchronously with the host genome through lysogenic cycles. The pre-ligase can mediate horizontal gene transfer, conferring phenotypic characteristics such as antibiotic resistance, environmental adaptability advantages, adhesion ability, or enhanced pathogenicity to the host bacteria. Prediction results revealed three intact prophage regions in the HGL1 genome, totaling 232,969 bp. The complete genome visualization map of the HGL1 strain was constructed using the Circos software (Fig. 3). The circular map displays the following contents from the outermost to the innermost circle: The outermost circle represents the genomic position coordinates; The second circle shows the COG functional classification annotation results of the genes; The third circle displays the distribution of ncRNAs; The fourth circle represents the genomic GC content—The inward blue segments represent regions where the GC content is below the genome-wide average, whereas the outward red segments represent regions where the GC content is above the genome-wide average.; The innermost circle shows the GC skew value—where inward-projecting green regions indicate that the G content is lower than the C content, and outward-projecting orange regions indicate that the C content is lower than the G content.

### Genomic functional annotation

Bioinformatics predictions show that the HGL1 strain genome encodes 43 proteins with signal peptides and 392 proteins with transmembrane domains. After comprehensive evaluation, 20 of them are identified as secretory proteins. Through functional database comparison analysis, 1484, 1032, 1115, 1186 and 596 genes were functionally annotated in the NR, GO, KEGG, COG and Swiss-Prot databases, respectively. Notably, the GO annotation count (4497) in Table 1 refers to the total number of assigned GO terms, as a single gene can be associated with multiple terms across the Biological Process, Cellular Component, and Molecular Function categories. The database with the fewest annotated genes is the CARD database with 4 genes.

**Table 1:** Overview of genome function analysis of strain HGL1

Database	Gene number
NR	1484
Swiss-Prot	596
KEGG	1115
COG	1186
GO	4497
PHI	146
Pfam	2972
VFDB	56
CARD	4
Secretory protein	455
T3SS	21
CAZY	73
TCDB	126

**Fig. 4:**
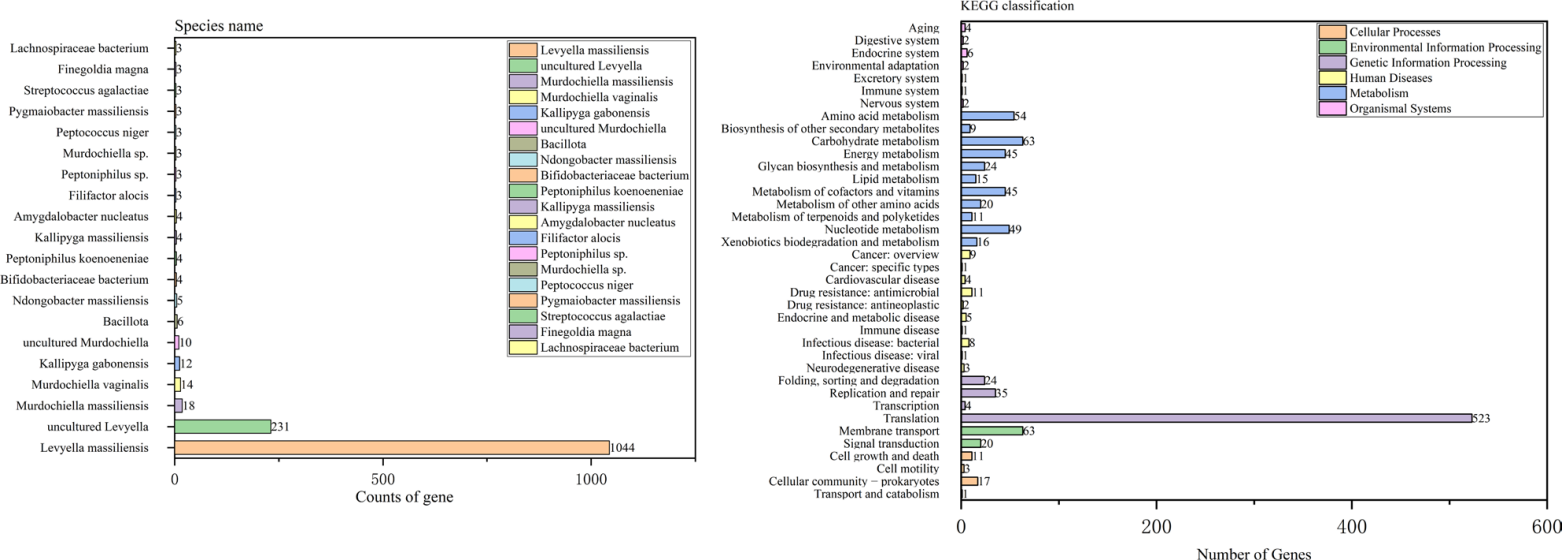
**A** NR analysis result of strain HGL1. **B** KEGG analysis for the result of strain HGL1

The annotation analysis based on the NR database revealed that a total of 1,484 genes in the HGL1 strain genome were functionally annotated. The distribution and quantity statistics of the species from which the gene annotations were derived are shown in Fig. 4A. Among them, *L. massiliensis* was the species with the highest number of annotations, accounting for 70.35%.

KEGG database annotation assigned 1,115 protein-coding genes to 38 metabolic pathways. The most enriched pathways were Translation (523 genes), Membrane Transport (63 genes), and Carbohydrate Metabolism (63 genes) (Fig. 4B), reflecting a genetic architecture centered on core cellular processes essential for growth and adaptation.

**Fig. 5:**
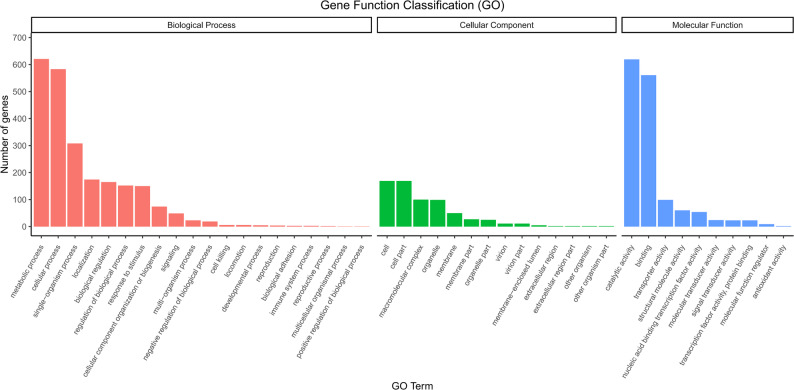
GO analysis for the result of strain HGL1

A total of 4,497 functionally annotated genes were identified through GO database analysis (Fig. 5), with metabolic process (621 genes) and cellular process (583 genes) being the categories containing the highest number of genes in biological processes; For cellular components, cell (169 genes) and cell part (169 genes) contained the highest numbers of annotated genes; while binding (619 genes) and catalytic activity (561 genes) were the most prominent functional terms in molecular functions.

**Fig. 6:**
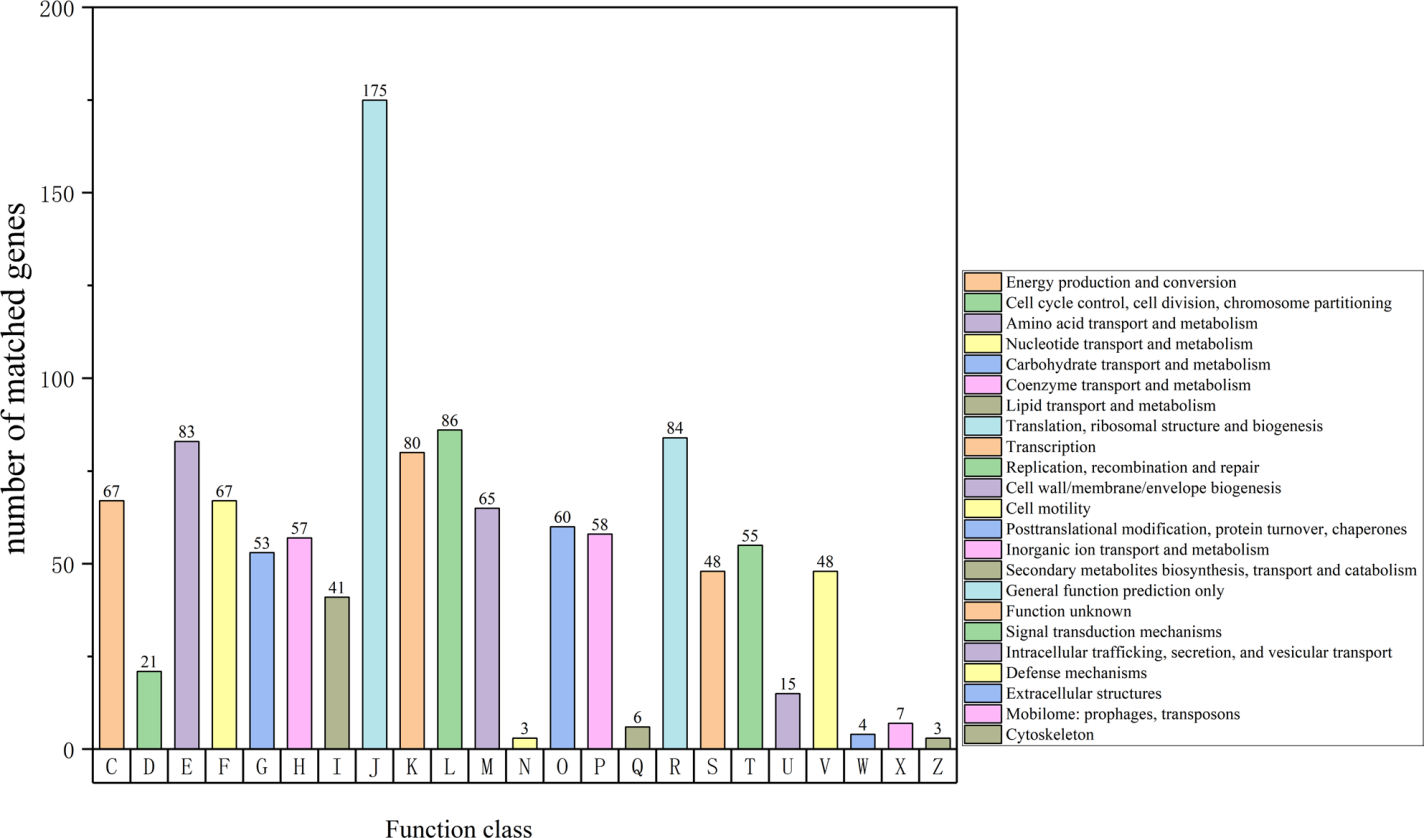
COG analysis for the result of strain HGL1

Functional annotation via the COG database identified 1,186 protein-coding genes, which were systematically classified into 23 functional categories (C-Z). The functional annotation results demonstrated that these genes were primarily involved in biological processes including: protein translation, cell membrane and envelope biogenesis, fundamental metabolism, amino acid metabolism, and transcriptional regulation (Fig. 6). These gene functions were predominantly associated with essential bacterial life activities, showing consistency with the aforementioned KEGG pathway analysis.

**Fig. 7:**
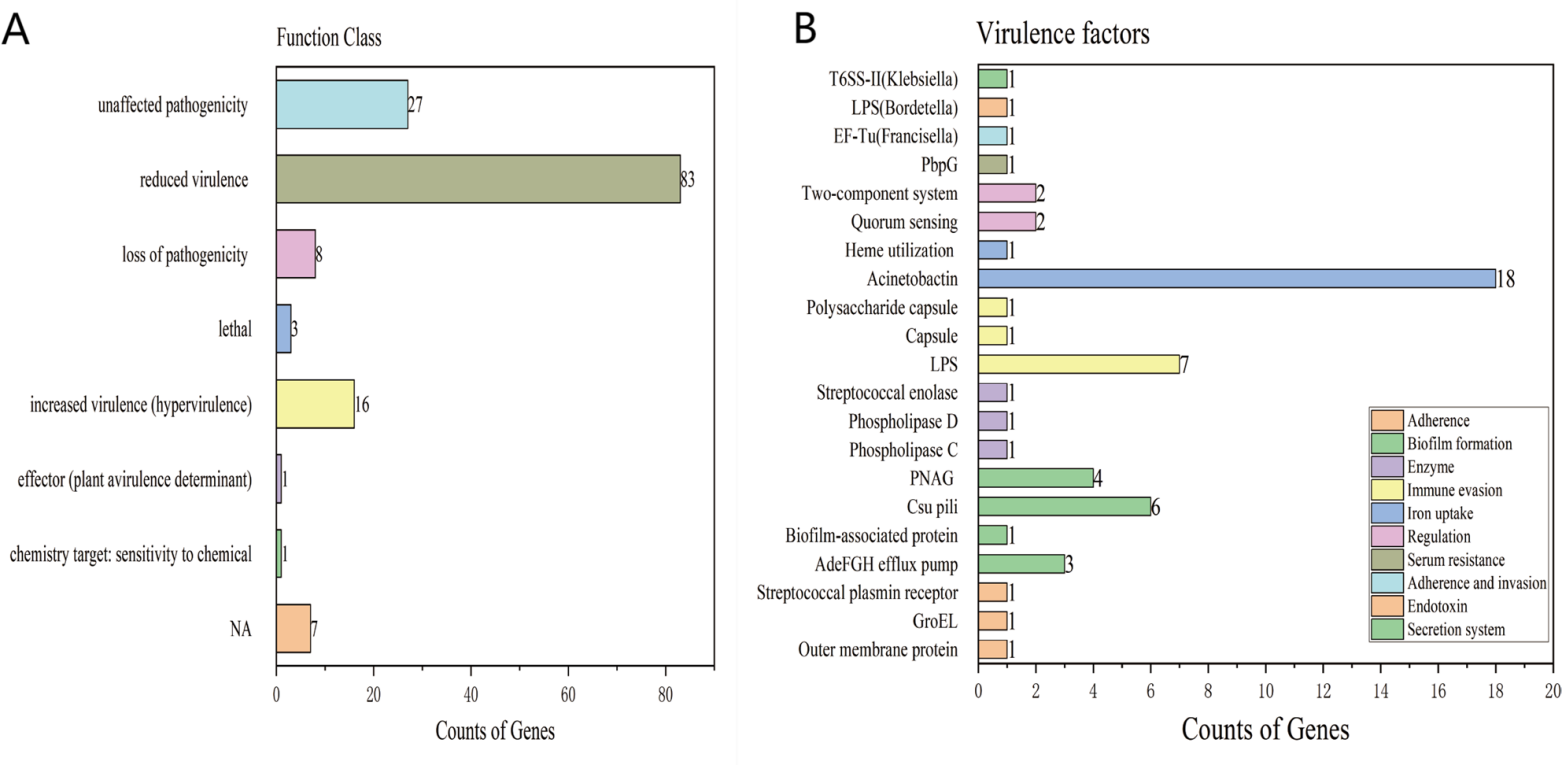
**A** Distribution map of types of pathogen-host interaction genes of strain HGL1. **B **VFDB analysis of strain HGL1

Analysis of pathogen-host interaction genes with the PHI database revealed that 146 annotated genes were classified into 8 categories, with “reduced virulence” (83 genes) being the largest group, followed by “unaffected pathogenicity” (27 genes) (Fig. 7A). The PHI database analysis further predicted the presence of 8 genes encoding putative effector proteins in the HGL1 genome. In bacterial pathogens, such effectors are typically secreted into the host environment or cells to manipulate host processes, which can facilitate bacterial adhesion, nutrient acquisition, and evasion of immune responses—key steps for successful colonization and infection.

This study compared the virulence factor database (VFDB) using the DIAMOND software. In strain HGL1, 56 virulence genes were identified (Figure 7B). The selection criteria were a sequence identity of ≥ 40% and an e-value of < 1e-10. The category with the largest number of annotated genes was *Iron uptake* (19 genes), followed by *Biofilm formation* (14 genes) and *Immune evasion* (7 genes). *Iron uptake* mainly includes *Acinetobactin* and *Heme utilization*. Among them, the *Acinetobactin*-related encoding genes were the most numerous, including *barA*,* basD*,* bauB and entE*, totaling 14 genes. *Biofilm formation* mainly consisted of *AdeFGH efflux pump/transport autoinducer*, *Biofilm*-associated protein, *Csupili* and *PNAG (Polysaccharide poly-N-acetylglucosamine)*. Through analysis using the CARD database, this strain carried a total of 4 antibiotic resistance genes: *tet(W)*,* ErmB*,* vanY* gene in the *vanF* cluster, and *AAC(6’)-Im*, which mediated *tetracycline* antibiotics and *macrolide* antibiotics, respectively. *lincosamide* antibiotic; Resistance to *streptogramin* antibiotics (A, B), *glycopeptide* antibiotics, and *aminoglycoside* antibiotics.

### Antimicrobial resistance of strainHGL1

The results of the antimicrobial susceptibility test and the minimum inhibitory concentration (MIC) determination for strain HGL are shown in Table 2. This strain shows resistance to the tested drugs such as *erythromycin*,* clarithromycin*,* azithromycin*,* trimethoprim/sulfamethoxazole*,* ceftriaxone*,* ciprofloxacin*,* levofloxacin*,* gentamicin*, and *Cefotaxime*, and is sensitive to *tigecycline*,* amoxicillin/clavulanic acid*, and *beta-lactam antibiotics.*

**Table 2:** Concentration of minimum growth inhibitory and antimicrobial susceptibility of strain HGL1

Antibiotics	Abbreviation	MIC	Interpretation
Erythromycin	E	≥ 256	R
Clarithromycin	CLR	≥ 256	R
Azithromycin	AZM	≥ 256	R
Trimethoprim/sulfamethoxazole	SXT	≥ 32	R
Ceftriaxone	CRO	≥ 32	R
Meropenem	MRP	0.19	S
Ampicillin	AMP	0.38	S
Ciprofloxacin	CIP	≥ 32	R
Levofloxacin	LEV	≥ 32	R
Gentamicin	CN	≥ 256	R
Chloramphenicol	C	3	I
Cefotaxime	CTX	24	R
Tigecycline	TIG	<=0.25	S
Polymyxin B	PB	<=0.25	S
Tetracycline	TET	<=1	S
Streptomycin	STR	<=4	S
Amoxicillin/Clavulanic Acid	AMC	<=1	S
Ertapenem	ETP	<=0.25	S
Imipenem	IPM	<=0.25	S

*S *Sensitive, *I *Intermediary, *R *Resistant

## Discussion

Anaerobic bacteria, as normal constituents of the human microbiota, can also act as opportunistic pathogens causing infectious diseases under certain conditions [21]. Although their exact clinical significance and true prevalence remain unclear, many anaerobic bacteria have been identified as causative agents of human infections such as bacteremia [22]. Since its formal designation in 2011 [6], research related to *L. massiliensis* has remained exceedingly limited. Beyond the initial report of its isolation from human samples and the acquisition of its 16 S rRNA gene sequence [6], investigations into its host range, pathogenic mechanisms, virulence factors, and antibiotic susceptibility are urgently needed.

In this study, we successfully isolated *L. massiliensis* for the first time from a blood sample of a patient in China, and preliminarily identified its taxonomic status through 16 S rRNA gene sequencing. Subsequently, we conducted systematic genomic functional and drug resistance analyses on the *L. massiliensis* strain. By completing whole-genome sequencing of this strain and performing functional annotation, we further extracted the 16 S rRNA sequence from its genome, and combined with the results of Average Nucleotide Identity (ANI > 95%) and digital DNA-DNA hybridization (dDDH > 70%), accurately identified this isolate as *L. massiliensis*. This discovery expands our understanding of the distribution range of *L. massiliensis* in humans, and provides important strain resources and a theoretical foundation for subsequent research on this bacterium. Furthermore, Annotation against the NR database revealed that approximately 70.35% of the predicted proteins from strain HGL1 have their closest homologs present in *L. massiliensis*, confirming a high degree of consistency at the species level. The remaining ~ 30% of genes are distributed across various other bacterial lineages, as shown in Fig. 3A. This phenomenon could have multiple explanations, but the more likely reasons include: (i) As a less-studied species, reference sequence information for *L. massiliensis* is limited. The NR database may lack complete genome data for this species, resulting in some genes failing to match this species; (ii) Bacterial genomes contain numerous conserved housekeeping genes as well as species-specific genes. Therefore, even though strain HGL1 belongs to L. massiliensis, some of its genes may still show homology with other bacterial lineages, reflecting the conserved and diverse nature of bacterial genomes.

Genomic annotation of the *L. massiliensis* strain revealed a series of genes associated with pathogenicity, including those involved in virulence enhancement, virulence diminution, and chemical sensitivity. Annotation results from GO, COG, and KEGG databases indicated that genes related to the maintenance of basic physiological functions were the most abundant in this bacterium, these genes collectively form the essential metabolic network required for its survival. Notably, 56 virulence genes were predicted in the VFDB database (Fig. 6B), these genes are distributed across various virulence factors including Acinetobactin, LPS, and the AdeFGH efflux pump, which may significantly enhance the bacterium’s resistance and pathogenic capabilities [23–26]. The PHI database primarily originates from fungal, oomycete, and bacterial pathogens, with infecting hosts encompassing animals, plants, fungi, and insects. This database plays a significant role in research aimed at identifying target genes for drug intervention, and it also includes antifungal compounds and their corresponding target genes. Each gene entry in the database includes nucleic acid and amino acid sequences, along with a detailed description of the predicted protein function during host infection. Annotation results of strain HGL1 against the PHI database (Fig. 6A) indicated the presence of 16 increased virulence (hypervirulence) genes and 3 lethal genes. These observations suggest that further monitoring and investigation of *L. massiliensis* may be warranted.

Secretory proteins are proteins synthesized within the cell and then secreted across the cell membrane to function extracellularly, guided by a signal peptide. Many secretory proteins are important enzymes essential for life processes. The N-terminus of a secretory protein consists of a signal peptide comprising 15–30 amino acids, which plays a leading role in its secretion. Pathogenic bacteria secrete such proteins into the extracellular environment or host cells via type N secretion systems (TNSS; currently, seven types are identified, types I–VII), eliciting pathological responses by manipulating immune responses and cellular apoptosis. This includes promoting adhesion to host cells and the extracellular matrix, facilitating invasion and intracellular survival, scavenging essential nutrients, disrupting host cell signaling and immune responses, and delivering toxins. In strain HGL1, we predicted 20 secretory proteins, 43 proteins with signal peptides, and 392 proteins with transmembrane domains. Among these, proteins destined for the extracellular space or outer membrane are of particular interest as primary candidates for host-pathogen interactions. Our annotation linked several of these to critical virulence-associated functions. Most notably, key components of the acinetobactin-like siderophore system (e.g., the biosynthetic proteins *BarA* and *BasD*, and the transport protein *BauB*) were predicted to contain signal peptides, consistent with their roles in iron acquisition from the host milieu. Similarly, some proteins associated with biofilm formation and efflux pump systems (e.g., components of the *AdeFGH* pump) were also predicted to be membrane-associated or secreted, aligning with their functions in surface adherence and antimicrobial resistance.

This study employed the E-test gradient concentration method to conduct antibiotic susceptibility testing on *L. massiliensis* HGL1. Results showed that this strain exhibited a multidrug-resistant phenotype, showing resistance to multiple drugs including *erythromycin*, *clarithromycin*, *azithromycin*, *trimethoprim/sulfamethoxazole*, *ceftriaxone*, *ciprofloxacin*, *levofloxacin*, *gentamicin*, and *cefotaxime*; but remained susceptible to *tigecycline*, *amoxicillin/clavulanic* acid, and some *β-lactam antibiotics*. Genomic analysis revealed that HGL1 carries 4 resistance genes, which are associated with mechanisms such as antibiotic target al.teration and antibiotic efflux [27–29], potentially representing the genetic basis for its resistant phenotype. The resistant phenotype of strain HGL1 to *trimethoprim-sulfamethoxazole*, third-generation cephalosporins (*ceftriaxone*,* cefotaxime*), and fluoroquinolones (*ciprofloxacin*,* levofloxacin*) cannot be explained by the genes annotated in the antibiotic resistance gene database (CARD). This “genotype-silent resistance” suggests the potential existence of unconventional resistance mechanisms. Possible mechanisms include: (i) mutations in chromosomal target genes (e.g., mutations in *gyrA* or *parC* genes conferring fluoroquinolone resistance); (ii) overexpression of intrinsic efflux pumps (for example, the *AdeFGH efflux pump* system annotated in this genome, whose upregulation can lead to broad-spectrum resistance to multiple drugs). Conversely, the presence of resistance genes alongside a susceptible phenotype is not uncommon in microbial genomes. Potential reasons include: the gene is not expressed (silent or lacking necessary activators); the gene itself carries inactivating mutations; These findings indicate that for understudied bacterial species, relying solely on existing databases for resistance prediction has significant limitations, and must be combined with phenotypic assays.

A comparative analysis was conducted with two previously reported cases of *L. massiliensis* infection, one involving multiple outpatient patients with acute leukemia (aged between 31 and 72 years) receiving chemotherapy, in whom the bacterium was detected in rectal swabs and fecal samples [7]; the other was a 72-year-old patient after inguinal hernia repair, with *L. massiliensis* detected in scrotal fluid from the surgical site [8]. Acute leukemia patients often have immunodeficiency due to lymphocyte abnormalities, making them more susceptible to opportunistic infections [30–33]; while surgical patients may experience decreased immune function postoperatively due to wounds and stress states [34–36]. Previously, the detection of *L. massiliensis* relied primarily on metagenomic signals in samples; consequently, its clinical significance and potential to cause invasive infections remained unclear. In this study, a viable strain of *L. massiliensis* (HGL1) was isolated from a sterile site (blood), providing new evidence that this bacterium can be present in the bloodstream under certain conditions. Combined with its multidrug-resistant phenotype and the presence of predicted virulence-associated factors in its genome (such as iron acquisition systems and biofilm formation), these findings suggest that *L. massiliensis* may be a potential opportunistic pathogen capable of causing invasive infections. Notably, in all reported cases (including the present one), detection of this bacterium has been associated with an immunocompromised status in patients, further suggesting a potential link with susceptible hosts. However, its exact role in the disease process—whether as a colonizer, a commensal, or a causative agent—remains to be elucidated through functional studies and further clinical observations. Furthermore, whether the genetic features identified in this study translate into actual pathogenic capacity in vivo also warrants further in vivo functional validation.

## Conclusion

In China, this study reports the first successful isolation of *L. massiliensis* from human blood samples and analyzes its morphological characteristics and phylogenetic position. Genomic and antimicrobial susceptibility testing results indicate that this strain possesses potential pathogenicity and a multidrug-resistant phenotype; however, the expression of these characteristics and their clinical significance require functional validation in host-relevant models. Future research should thoroughly investigate its pathogenic mechanisms under conditions that simulate the host environment. Our findings also suggest the need to enhance clinical monitoring and antimicrobial susceptibility data sharing for this type of anaerobic bacteria, to improve the accuracy and effectiveness of infection diagnosis and treatment.

## Data Availability

The complete genome data of Strain HGL1 set was submitted to the National Biotechnology Information Center (NCBI) database (Accession number: CP195136). Data has been uploaded to the website ( *https://www.ncbi.nlm.nih.gov/bioproject/PRJNA1271292* ). The 16 S rRNA gene sequence of Strain HGL1 was submitted to the GenBank (Accession number: PV731429). Data has been uploaded to the website ( *https://www.ncbi.nlm.nih.gov/nuccore/PV731429* ). We have uploaded the raw sequencing data to the SRA database of NCBI. The accession numbers are: SRR35756613 (PacBio: *https://www.ncbi.nlm.nih.gov/sra/SRR35756613* ) andSRR35756614 (Illumina: *https://www.ncbi.nlm.nih.gov/sra/?term=SRR35756614* ).

## Abbreviations

ANI: Average Nucleotide Identity
dDDH: Digital DNA-DNA hybridization
MALDI-TOF MS: Matrix-Assisted Laser Desorption/Ionization Time-of-Flight Mass Spectrometry
ATCC: American Type Culture Collection
